# Response: Commentary: Modeling mortality risk in patients with severe COVID-19 from Mexico

**DOI:** 10.3389/fmed.2023.1301349

**Published:** 2023-11-21

**Authors:** Gerald Stanley Zavorsky, Arturo Cortes-Telles, Esperanza Figueroa-Hurtado, Diana Lizbeth Ortiz-Farias

**Affiliations:** ^1^Department of Physiology and Membrane Biology, University of California, Davis, Davis, CA, United States; ^2^Respiratory and Thoracic Surgery Unit, Hospital Regional de Alta Especialidad de la Península de Yucatan, Yucatan, Mexico

**Keywords:** LASSO, least absolute shrinkage and selection operator, COVID-19, mechanical ventilation, biostatistics

We appreciate the commentary from Sanjari et al. (1) about our recent work (2). We want to respond:

A. Large odds ratios are not necessarily the best predictors

They mention that sizable odds ratios don't always pinpoint the top predictor, suggesting that we should be reporting the area under the receiver operating characteristic curve (AUC). We concur that a variable with the highest odds ratio in regression can indicate a strong association but might not be the most important predictor. To gauge how the model fits to the outcome (1 = death, 0 = alive at discharge), we can separately assess the Bayesian Information Criterion (BIC) for each predictor. These predictors can be arranged in order of significance based on the BIC: the lowest BIC is the most important, while the highest BIC points to the least important in the model.
In our study, the predictor boasting the largest odds ratio, which was the requirement for mechanical ventilation (1 meaning yes, 0 meaning no), was also the one with the smallest BIC (BIC = 153.7; log-likelihood = −71.4) when stacked against the other four predictors in our model. Following that, the second most vital predictor when analyzed individually was the pulse oximetry saturation upon admission (BIC = 258.8; log-likelihood = −123.9), succeeded by the logarithm of the derived neutrophil-to-lymphocyte ratio (dNLR; BIC = 296.9; log-likelihood = −142.9), age (BIC = 307.9; log-likelihood = −148.4), and lastly, the logarithm of platelet counts upon admission (BIC = 340.9 and log-likelihood = −164.9). Additionally, a “random forests” visualization (3) demonstrated that the need for mechanical ventilation emerged as the paramount predictor among the five. This conclusion was drawn from the marked decrease in accuracy in our model presented in Table 3 of our publication when this predictor was switched out randomly. The significance rank of the subsequent four variables aligned with their BIC rankings.
In the discussion segment of our paper (2), we highlight that the AUC of a receiver operating characteristic should no longer be used as an indicator for screening performance (4). The AUC's value isn't intuitive for many; for instance, an AUC of 0.70 doesn't immediately provide insights into the detection rate (also known as sensitivity) for a given rate of false positives (which is the inverse of specificity), or the other way around. Furthermore, when standard deviations of screening markers vary between affected and unaffected subjects, the AUC can become ambiguous or even give a false impression. Matthew's correlation coefficient offers a more accurate approach for classification (5–7).

B. LASSO is not appropriate for explanation modeling

In our paper's methods section (2), we employed LASSO for pinpointing the key predictors linked to mortality. LASSO not only selects essential variables but also reduces other coefficients to zero, effectively guarding against model overfitting. This approach melds both variable selection and regularization into a robust regression methodology. After applying LASSO logistic regression, we transitioned to standard logistic regression for the identified predictors. This step was taken to offer readers clear coefficient estimates, enabling them to assess the predictors without the influence of the L1 penalty. Indeed, as seen in Table 3 of our study (2), all five predictors had a Variance Inflation Factor under 2.0, indicative of minimal multicollinearity.

C. The presence of sparse data bias

Our study encompassed 247 participants (2). Out of these, 138 (or 56%) had to undergo mechanical ventilation. Furthermore, 101 out of the 247 individuals, accounting for 40%, unfortunately passed away. Notably, there wasn't any instance of variables with scant observations, ensuring data robustness.
Sanjari et al. (1) state that due to the exponentiation of some of our coefficients, there were enormous odds ratios, specifically for baseline platelet counts and dNLR. Due to the evident skewness in these values (1.6 and 4.5 respectively), we employed logarithmic transformations on these predictors to mitigate the impact of potential outliers. As a result, this adjustment offered a more harmonized model fit, as indicated by the Bayesian Information Criterion (BIC). When comparing our chosen model showcased in Table 3 of our publication (2) to its counterpart without the logarithmic adjustments on platelets and dNLR, the evidence ratio stands at 1.44. This suggests that the data backs our presented model in Table 3 approximately 1.44 times more than the alternative model. It's an indication that our model choice is on the right track.
Yet, in the spirit of complete disclosure, when we exhibit the model analogous to what's in Table 3 of our paper (2) but without logarithmic adjustments for the baseline platelet counts or dNLR, the odds ratios for age (1.07, 95% CI = 1.03–1.12), pulse oximetry saturation during admission (0.96, 95% CI = 0.92–0.997), and the need for mechanical ventilation (164, 95% CI = 39–700) hold steady. Breaking it down: each yearly increase in age increases the odds of death by 3% to 12%; each 1% elevation in pulse oximetry saturation at the point of admission curtails the odds of death by 0.3%−8%; and the necessity for mechanical ventilation amplifies the odds of death from 39 to as much as 700 times. When it comes to baseline platelet counts (expressed as × 10^9^ cells/L), the odds ratio was 0.99 (95% CI = 0.985–0.997), and for the baseline dNLR, it was 1.21 (95% CI = 1.04–1.39). Hence, with every increment of 1 × 10^9^ cell per liter in platelets, mortality odds are reduced by 0.3%−1.5%. On the flip side, every unit hike in dNLR pushes the death odds up by 4%−39%.

Firth's bias-reduced regression (8) is a nice thought, but implementing this statistical technique resulted in coefficients like our model presented in Table 3. We used the R “*logistic*” package (version 1.26.0), and there was no practical difference between the model result from Firth's biased reduced regression and our model in Table 3.

We appreciate the commentary by Sanjari et al. (1) concerning our paper highlighting our contribution to the field.

## Author contributions

AC-T: Writing – review & editing. EF-H: Writing – review & editing. DO-F: Writing – review & editing. GZ: Formal analysis, Methodology, Writing – original draft, Writing – review & editing.
